# Dwell Time and Risk of Bloodstream Infection With Peripheral Intravenous Catheters

**DOI:** 10.1001/jamanetworkopen.2025.7202

**Published:** 2025-04-24

**Authors:** Marie-Céline Zanella, Gaud Catho, Holly Jackson, Nasim Lotfinejad, Valérie Sauvan, Marie-Noëlle Chraiti, Walter Zingg, Stephan Harbarth, Niccolò Buetti

**Affiliations:** 1 Infection Control Program and World Health Organization Collaborating Centre, Faculty of Medicine, Geneva University Hospitals, Geneva, Switzerland; 2 Department of Infectious Diseases and Hospital Epidemiology, University Hospital Zurich, Zurich, Switzerland; 3 INSERM, IAME, Université Paris–Cité, Paris, France

## Abstract

**Importance:**

Bloodstream infections (BSIs) associated with peripheral intravenous catheters (PIVCs) are rare but preventable adverse events. The association of dwell time with the risk of BSIs with PIVCs remains controversial.

**Objective:**

To analyze the risk of BSIs during PIVC maintenance therapy.

**Design, Setting, and Participants:**

In this observational cohort study, all patients hospitalized at Geneva University Hospitals with at least 1 PIVC insertion on the upper extremity (N = 371 061) between January 1, 2016, and February 29, 2020, were evaluated. Statistical analysis was performed from January 2023 to January 2025.

**Exposure:**

At least 1 PIVC insertion on the upper extremity.

**Main Outcomes and Measures:**

The primary outcome was BSIs with PIVCs; data were collected by prospective BSI surveillance. The daily risk of BSIs with PIVCs was analyzed using the hazard rate function by kernel-based methods. Multivariable logistic models were performed to evaluate the risk of BSIs with PIVCs comparing different cutoff values of dwell times (>3 vs ≤3 days, >4 vs ≤4 days, >5 vs ≤5 days, and >6 vs ≤6 days).

**Results:**

A total of 371 061 PIVCs (median patient age, 63 years [IQR, 41-79 years]; 187 786 women [51%]) with documented catheter duration were included. A total of 140 178 PIVCs (38%) had a dwell time of 1 to 2 days, 119 252 (32%) had a dwell time of 3 to 4 days, and 111 631 (30%) had a dwell time of more than 4 days. The instantaneous risk of BSIs with PIVCs was low in the first 2 days of dwell time and increased rapidly thereafter. The risk of BSIs was significantly increased after 3 days of catheter maintenance (adjusted odds ratio [AOR], 13.55; 95% CI, 5.44-34.00). This risk was the highest after 3 days and remained increased thereafter (>4 days: AOR, 8.53; 95% CI, 4.47-16.28; >5 days: AOR, 5.38; 95% CI, 3.23-8.96; and >6 days: AOR, 7.63; 95% CI, 4.57-12.74).

**Conclusions and Relevance:**

In this cohort study of 371 061 PIVCs, dwell time was associated with the development of BSIs with PIVCs. After day 3, PIVC indication should be reviewed and PIVC replacement considered.

## Key Points

**Question:**

What is the association of dwell time with the risk of bloodstream infections with peripheral intravenous catheters (PIVCs)?

**Findings:**

In this cohort study conducted at Geneva University Hospitals and including 371 061 PIVCs, the risk of bloodstream infections was significantly increased after 3 days of dwell time.

**Meaning:**

This study suggests that the indication for PIVC should be challenged and PIVC replacement should be considered after 3 days of dwell time.

## Teaser

This cohort study analyzes the association of dwell time with the risk of bloodstream infections during peripheral intravenous catheter maintenance therapy.

## Introduction

Peripheral intravenous catheter (PIVC) insertions are frequent among hospitalized patients. A global audit across 13 countries showed that almost 60% of patients had at least 1 PIVC in situ.^1^ A point-prevalence study in Switzerland illustrated a similar prevalence, with 49% of patients having a PIVC.^2^ The most frequently reported complications associated with PIVCs are phlebitis, hematoma, and extravasation.^3^ Few data are available on the risk of bloodstream infections (BSIs) associated with PIVCs. A systematic review showed that the risk of BSIs with PIVCs may be underestimated.^4^ Moreover, BSIs with PIVCs are associated with increased mortality and are therefore an important outcome to evaluate PIVC-associated risks.^5^ In addition, infection-related costs are substantial.^6,7^

The role of dwell time in the development of BSIs with PIVCs remains debated.^4^ In 2019, a systematic review and meta-analysis illustrated that routine replacement (vs clinically indicated replacement) of PIVCs was not associated with BSIs.^8^ However, a recent study challenged these results in a large cohort study with a before-after design including more than 400 000 PIVCs.^9^ The study demonstrated that a clinically indicated replacement of PIVCs was associated with an increased risk of BSIs. Here, using the same high-quality database, we analyzed the risk of BSIs with PIVCs during catheter maintenance therapy.

## Methods

### Setting, Patients, and PIVCs

We performed a cohort study at Geneva University Hospitals (HUG), the largest tertiary care center network in Switzerland, which includes 5 rehabilitation and/or palliative care sites, 1 acute care site, 1 geriatrics site, 1 pediatrics site, 1 gynecology-obstetrics site, and 1 psychiatry site. HUG has approximately 2100 beds and receives 60 000 hospital admissions per year. All pediatric and adult hospitalized patients with at least 1 PIVC insertion on the upper extremity between January 1, 2016, and February 29, 2020 (ie, start of the COVID-19 pandemic), were included. Peripheral intravenous catheters inserted outside of HUG and PIVCs inserted in the lower extremity were excluded. Peripheral intravenous catheters with secured injection sites were used and midlines were not inserted at HUG during the study period. Hospital-acquired BSI surveillance is part of a mandatory indicator surveillance at HUG and thus is considered as quality assurance. Only pseudonymized data have been processed; therefore, ethical board approval for data reuse is not required in accordance with HUG policy. This study followed the Strengthening the Reporting of Observational Studies in Epidemiology (STROBE) reporting guideline.^10^

### Data Sources

We extracted data on ward type, patient characteristics (sex, age, and dates of admission and discharge on the ward where the PIVC was inserted), and PIVC characteristics (dwell time and insertion site) from the electronic health record system. Data on BSIs with PIVCs (date of onset, ward attribution, microorganism) were collected from the hospital-wide BSI surveillance, which has been performed prospectively by the Infection Control Program of HUG for over 25 years.

### Definitions

The definition of the primary outcome, BSI with PIVC, was based on the European Centre for Disease Prevention and Control definition^11^: a positive blood culture occurring from catheter insertion until 48 hours after catheter removal, with the same microorganism as isolated from a positive superficial culture from purulent exudate from the insertion site or a quantitative PIVC tip culture of 10^3^ colony-forming units/mL or more. Catheter tip cultures were not routinely performed at HUG. Alternatively, a positive blood culture occurring from the day of insertion until 48 hours after catheter removal, resolution of symptoms within 48 hours after catheter removal, and the absence of any other infectious focus was defined as BSI with PIVC as well. Common skin contaminants were considered relevant only if the patient had at least 1 sign or symptom of infection (chills, hypotension, or fever [>38 °C]) and at least 2 positive blood cultures from 2 separate blood samples within 48 hours. Common skin contaminants included coagulase-negative staphylococci, *Bacillus* spp, *Propionibacterium* spp, *Corynebacterium* spp, and *Micrococcus* spp

### Infection Prevention and Control Procedures

From January 2016 to February 2020, the infection control practices for PIVC insertion and maintenance care were the following: (1) the choice of the insertion site was left at the discretion of the nurse or physician; (2) PIVC insertion using ultrasonography-guided assistance was not routinely performed during the study period; (3) only alcohol-based 2% chlorhexidine gluconate was available for skin antisepsis on catheter insertion and dressing change; (4) semipermeable transparent dressings were used consistently and were changed when clinically indicated (soiled, leaking, or wet dressings were required to be immediately replaced); (5) the insertion site was required to be inspected daily for clinical signs, indicating malfunctioning or signs of phlebitis or local infection; and (6) systematic sterile flushing after product administration was routinely performed. All PIVCs inserted at HUG are standard catheters (as opposed to integrated catheters).

Peripheral intravenous catheters were routinely replaced every 4 days from January 1, 2016, to March 31, 2018, and from October 16, 2019, to February 29, 2020. From April 1, 2018, to October 15, 2019, PIVCs were replaced if clinically indicated.^9^

### Statistical Analysis

Statistical analysis was performed from January 2023 to January 2025. We described characteristics of PIVCs as counts and percentages for categorical variables or median (IQR) values for numeric variables. The statistical plan had 5 steps. First, we illustrated the number of PIVCs included and BSIs with PIVCs in situ at different dwell times. Second, the daily risk of BSIs with PIVCs was analyzed using the hazard rate function by kernel-based methods, where time was taken as a continuous variable.^12^ Third, we described the characteristics of included PIVCs with different dwell times (1-2 days, 3-4 days, and >4 days). Fourth, to investigate when PIVC replacement should be considered, we evaluated the risk of BSIs with PIVCs before vs after different cutoff values of dwell times, using multivariable logistic models. The main variable of interest, dwell time, was dichotomized by selecting a cutoff value and inputted into our multivariable models; other variables, which may be associated with the risk of BSIs with PIVCs, were used as adjustment factors (ie, sex, age, time from hospital admission to PIVC insertion, and insertion site). Several analyses were performed considering different cutoff values (>3 vs ≤3 days, >4 vs ≤4 days, >5 vs ≤5 days, and >6 vs ≤6 days). In addition, we performed several sensitivity analyses. We used inverse probability–weighted logistic models (using the covariates age, sex, insertion site, and time from hospital admission to catheter insertion) to investigate the association between different dwell time cutoffs and BSIs with PIVCs (eMethods in Supplement 1). We performed a sensitivity analysis using only PIVCs included during the “replaced if clinically indicated” period (from April 1, 2018, to October 15, 2019). The analyses assumed independence between PIVCs. To mitigate the bias introduced by multiple PIVC insertions for an individual patient and because a random-effect models did not converge due to the low number of BSIs with PIVCs, we performed a sensitivity analysis limiting only to the first PIVC inserted for a patient.

Fifth, to evaluate the infectious risk for different dwell times, we performed additional analyses using multivariable logistic models (adjustment factors were sex, age, time from hospital admission to PIVC insertion, and insertion site), considering the dwell time as a categorical variable (4 days, 5 days, ≥6 days) and using 3 days or less as reference.

Tests were 2-tailed, with *P* < .05 being considered statistically significant. We performed all analyses using SAS, version 9.4 (SAS Institute Inc).

## Results

### Patients, BSIs With PIVCs, and Dwell Time

**Figure 1.**
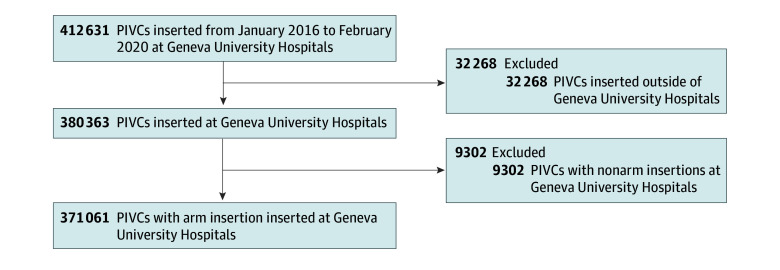
Study Flowchart PIVC indicates peripheral intravenous catheter.

After exclusion of 32 268 PIVCs inserted outside of HUG and 9302 PIVCs with nonarm insertions, a total of 371 061 PIVCs (median patient age, 63 years [IQR, 41-79 years]; 187 786 women [51%] and 183 275 men [49%]) were included (Figure 1; Table). The mean (SD) number of PIVCs per patient stay was 1.7 (1.6), and PIVCs were frequently inserted at the first hospitalization day (day 1; IQR, 1-5 days). Characteristics of patients with PIVCs with different dwell times are illustrated in the Table.

**Table.** Characteristics of the Study Population and PIVCs Inserted From January 2016 to February 2020

Characteristic	All PIVCs inserted (N = 371 061)^a^	PIVCs with dwell time of 1-2 d (n = 140 178)	PIVCs with dwell time of 3-4 d (n = 119 252)	PIVCs with dwell time of >4 d (n = 111 631)
Sex				
Female	187 786 (51)	74 063 (53)	62 322 (52)	51 401 (46)
Male	183 275 (49)	66 115 (47)	56 930 (48)	60 230 (54)
Age, median (IQR), y	63 (41-79)	54 (35-73)	62 (40-78)	72 (55-83)
ICU	9152 (3)	4000 (3)	2877 (2)	2275 (2)
Time from hospital admission to catheter insertion, median (IQR), d	1 (1-5)	1 (1-2)	1 (1-5)	2 (1-8)
Catheter-days, median (IQR), d	3 (2-5)	2 (1-2)	3 (3-4)	5 (5-7)
Insertion site				
Forearm	187 726 (51)	62 983 (45)	60 120 (50)	64 623 (58)
Arm	9642 (3)	2578 (2)	2917 (2)	4147 (4)
Antecubital fossa	39 375 (11)	18 775 (13)	10 374 (9)	10 226 (9)
Hand	92 585 (25)	39 336 (28)	32 035 (27)	21 214 (19)
Wrist	41 733 (11)	16 506 (12)	13 806 (12)	11 421 (10)
BSI with PIVC^b^	61 (0.02)	2 (0.001)	10 (0.01)	49 (0.04)

Abbreviations: BSI, bloodstream infection; ICU, intensive care unit; PIVC, peripheral intravenous catheter.

^a^ Patients may have had multiple PIVCs inserted.

^b^ Overall, we observed a total of 7 of 61 BSIs associated with PIVCs (12%), defined as a positive blood culture occurring from catheter insertion until 48 hours after catheter removal, with the same microorganism as isolated from a positive superficial culture from purulent exudate from the insertion site or a quantitative PIVC tip culture of 10^3^ colony-forming units/mL or more (see Methods).

**Figure 2.**
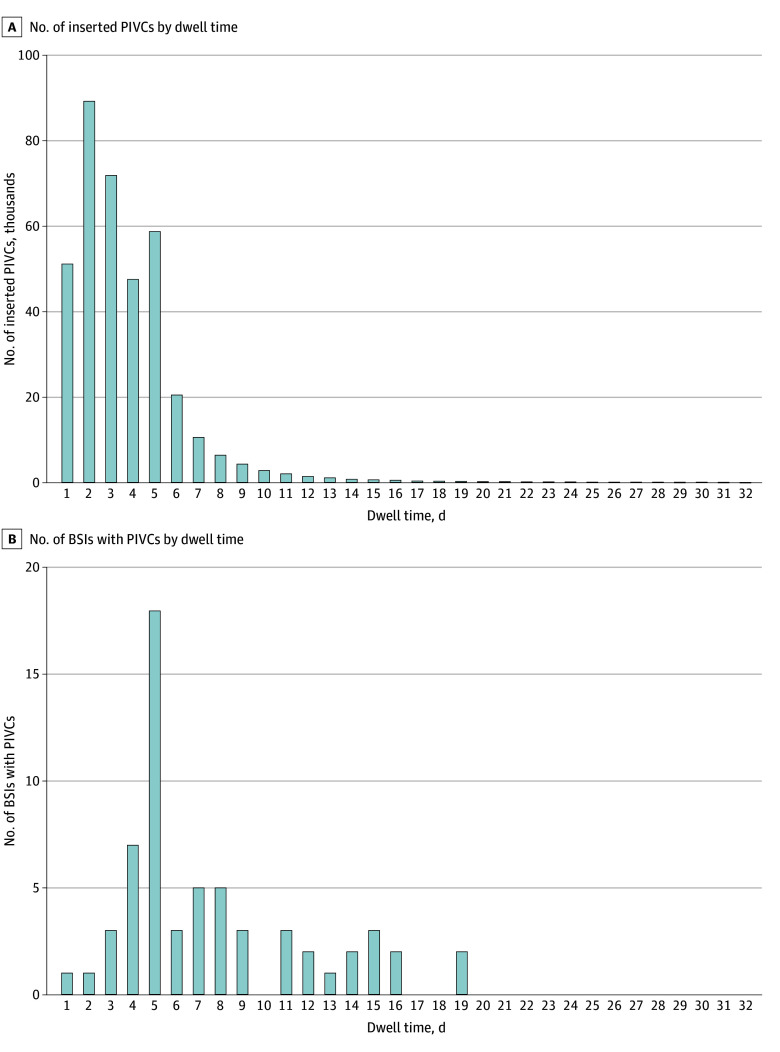
Number of Peripheral Intravenous Catheters (PIVCs) Inserted and Number of Bloodstream Infections (BSIs) With PIVCs According to Dwell Time

Peripheral intravenous catheters mostly remained in situ until day 5; the number of inserted PIVCs decreased thereafter (Figure 2A). A total of 140 178 PIVCs (38%) had a dwell time of 1 to 2 days, 119 252 (32%) had a dwell time of 3 to 4 days, and 111 631 (30%) had a dwell time of more than 4 days (Table). Peripheral intravenous catheters were inserted mostly in the forearm (187 726 [51%]). Sixty-one BSIs with PIVCs were observed during the study period. All BSIs with PIVCs were observed among adult patients. The number of BSIs with PIVCs was the highest in PIVCs removed at day 5 (Figure 2B).

**Figure 3.**
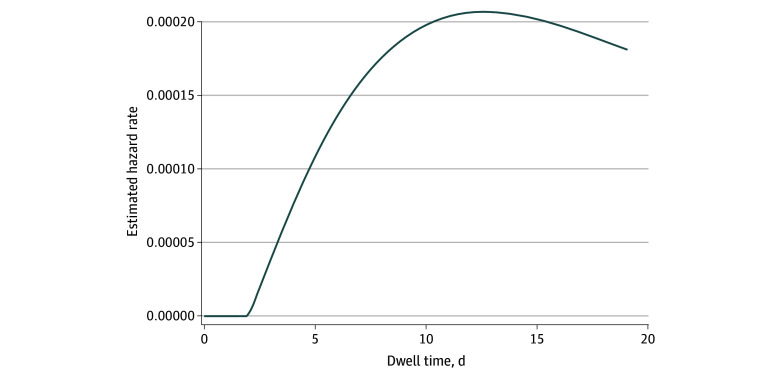
Instantaneous Hazard of Bloodstream Infections (BSIs) With Peripheral Intravenous Catheters (PIVCs)

As shown in Figure 3, the instantaneous hazard of PIVC-BSI was low during the first 2 days of catheter maintenance and increased rapidly until day 11. Only 6534 PIVCs had a dwell time of more than 11 days.

Peripheral intravenous catheters with a dwell time of more than 4 days were less frequently inserted among females (51 401 of 111 631 [46%]) and more frequently inserted among older patients (median age, 72 years [IQR, 55-83 years]) (Table). The median time from hospital admission to catheter insertion was longer for PIVCs in situ more than 4 days (2 days [IQR, 1-8 days]) compared with PIVCs with shorter dwell times. Peripheral intravenous catheters with a dwell time of more than 4 days were less frequently inserted in the hand.

### Multivariable Logistic Models

**Figure 4.**
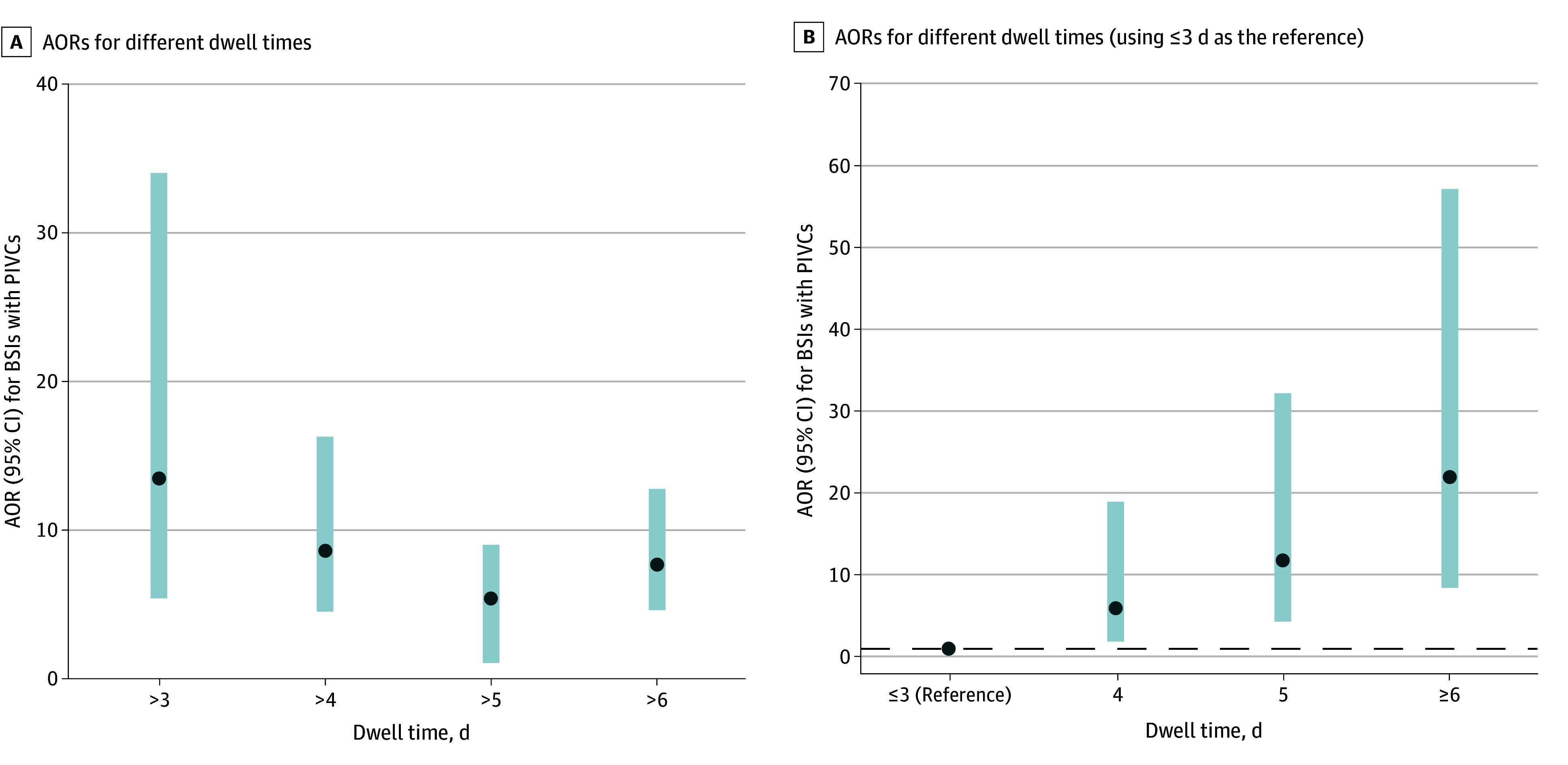
Adjusted Odds Ratios (AORs) for Different Dwell Times A, AOR for different dwell times (x-axis: reference for >3 days was ≤3 days, for >4 days was ≤4 days, for >5 days was ≤5 days, and for >6 days was ≤6 days). B, AOR for different dwell times, using dwell times of 3 days or less as reference. The dashed line represents the category of reference. BSI indicates bloodstream infection; and PIVC, peripheral intravenous catheter.

Multivariable logistic models showed that confidence intervals for odds ratios (ORs) were high comparing dwell times of 3 days or less with dwell times of more than 3 days and decreased with longer dwell times (Figure 4A). A high risk of BSIs with PIVCs was observed after 3 days (vs ≤3 days) of dwell time (adjusted OR [AOR], 13.55; 95% CI, 5.44-34.00; *P* < .001) (eTable 1 in Supplement 1) and remained increased thereafter. An increased risk was also observed after 4 days (vs ≤4 days) of dwell time (AOR, 8.53; 95% CI, 4.47-16.28), after 5 days (vs ≤5 days) of dwell time (AOR, 5.38; 95% CI, 3.23-8.96), and after 6 days (vs ≤6 days) of dwell time (AOR, 7.63; 95% CI, 4.57-12.74; *P* < .001) (eTable 2 in Supplement 1). The sensitivity analysis using propensity score revealed similar results for the different dwell times (eTable 3 and eFigure in Supplement 1). A sensitivity analysis including only the first catheter during hospitalization (n = 223 882) showed similar results after 3 days of catheter maintenance (AOR, 16.26; 95% CI, 4.86-54.46; *P* < .001) (eTable 4 in Supplement 1). A sensitivity analysis including only PIVCs inserted during the “replaced if clinically indicated” period (n = 117 620) showed similar results after 3 days of dwell time (OR, 18.42; 95% CI, 5.66-60.01; *P* < .001).

Additional analyses using multivariable logistic models using the dwell time as a categorical variable (≤3 days as a reference) showed a high risk of BSIs for PIVCs with a dwell time of 4 days (AOR, 6.02; 95% CI, 1.91-19.04) and an increasing risk at day 5 (AOR, 11.86; 95% CI, 4.34-32.39) and 6 days or more (AOR, 22.06; 95% CI, 8.48-57.39) (Figure 4B; eTable 5 in Supplement 1).

### Microbiology

Forty-nine BSIs with PIVCs (80%) occurred after day 4. We did not observe a significant difference in microbiologic cause between 4 days or fewer and more than 4 days of dwell time. However, 10 of the 49 BSIs with PIVCs (20%) with a dwell time of more than 4 days were due to *Staphylococcus aureus* (eTable 6 in Supplement 1).

## Discussion

Using a large prospective database and including more than 370 000 PIVCs, we found that the dwell time is associated with the development of BSIs with PIVCs. The infectious risk increased sharply at the third insertion day and remained high compared with short dwell times. Most BSIs with PIVCs occurred after 4 days of dwell time.

Probably due to their low incidence, BSIs with PIVCs are rarely investigated and only few studies have focused on this outcome, which is associated with increased mortality.^13^ However, considering the widespread use of PIVCs among hospitalized patients, the burden of BSIs with PIVCs is not negligible. There is a need for more high-quality studies on effective preventive measures for PIVC management.^14^ The World Health Organization has recently published guidelines for prevention of BSIs with PIVCs.^15^ Regarding the risk of BSIs with PIVCs based on PIVC dwell time, data in the literature are scant and controversial. One study that included surgical patients with a PIVC showed that there was no difference in dwell time among those with and without a BSI.^16^ Another study from the 1980s observed that among 17 BSIs with PIVCs with known duration of catheterization, 15 episodes involved PIVCs in place 3 or more days.^17^ In 1994, Fry et al^18^ found that almost 50% of BSIs with PIVCs occurred with dwell times of more than 4 days. A monocentric study including PIVCs from 2002 to 2006 showed that two-thirds of BSIs with PIVCs involved PIVCs with a dwell time of 4 days or more.^19^ In 2013, Freixas et al^20^ showed that among intravascular catheter infections originating from PIVCs, more than 60% occurred 72 hours after catheter insertion. On one hand, these analyses were mostly monocentric, retrospective, and conducted in the 1980s and 1990s, likely representing studies performed in the era before infection preventive bundles were routinely implemented. In this context, it is conceivable that most current state-of-the-art prevention measures were not routinely implemented.^21^ On the other hand, data from randomized clinical trials that compared routine replacement vs clinically indicated replacement did not provide relevant answers. Indeed, they did not assess the daily risk of infection due (1) to the low number of BSIs with PIVCs included and (2) to the difficulty to randomize patients with different dwell times.^8^ In an environment where BSIs with PIVCs were prospectively followed up, probably representing one of the largest datasets assembled, we founf that dwell time was associated with the occurrence of BSIs with PIVCs. We observed that the daily risk increased constantly after 2 days of catheter maintenance, thus highlighting the need of strong prevention measures (ie, routine replacement) to avoid infectious complications. We observed the highest risk of BSIs with PIVCs for dwell times of more than 3 days compared with shorter dwell times and found that 80% of BSIs with PIVCs occurred after day 4. We observed that coagulase-negative staphylococci were the most frequently identified microorganisms for PIVCs with dwell time of more than 4 days, as well as a trend toward an increased proportion of *S aureus* BSIs for PIVCs with dwell time of more than 4 days. We also observed a similar proportion of gram-negative bacteria for PIVCs with a dwell time of 4 days or fewer and a dwell time of more than 4 days.

Our results have important clinical implications; it is conceivable that after day 3, the indication of PIVC maintenance therapy should be challenged and PIVC replacement at least considered and, at day 4, replacement should be routinely performed. Other preventive measures aimed at reducing extraluminal PIVC contaminations (eg, antimicrobial impregnated dressing) and allowing prolonged PIVC catheterization could be considered. However, to our knowledge, such additional prevention strategies were investigated only in peripheral arterial catheters or central venous catheters and are not currently recommended for PIVCs.^22^

### Limitations

Our study has several limitations. First, this was a single-center study, limiting the generalizability of the results; however, HUG is composed of several different sites, thus increasing the diversity of the patient population. Second, we conducted an observational study without access to some clinical data (eg, disease severity, experience of the operator); however, we adjusted our analyses for several covariates that were electronically extracted. Third, due to the low incidence of BSIs with PIVCs, mixed logistic models, which take into account several PIVCs for an individual patient, did not converge. Therefore, all of our models assumed independence between PIVCs. To reduce this potential bias, we performed a sensitivity analysis that considered only the first PIVC inserted during the hospitalization, which showed similar results. Fourth, our models did not consider other important outcomes that may compete with BSIs with PIVCs (eg, mortality and phlebitis). Fifth, we did not observe BSIs with PIVCs among pediatric patients, and our results can be generalized only to the adult population. Sixth, further cost-effectiveness analyses should be conducted to provide more information about the ideal time at which a PIVC should be replaced.

## Conclusions

The results of this cohort study using a large prospective surveillance database suggest that dwell time is associated with the development of BSIs with PIVCs. The infectious risk increased after 3 days of dwell time compared with shorter catheter duration and most BSIs were identified after 4 days of dwell time. Based on these results, PIVC maintenance therapy should be challenged at day 3 and PIVC replacement should be at least considered; PIVC replacement should be performed at day 4.

## Notes

Supplement 1.
eMethods.
**eTable 1.** Multivariable Logistical Models Adjusting for Age, Sex, Time to Catheter Infection and Insertion Site **eTable 2.** Multivariable Logistical Models Accounting for Different Dwell Times **eTable 3.** Inverse Probability Treatment Weighted Logistical Regression Models for Different Dwell Times **eTable 4.** Multivariable Logistical Models Considering the First Catheter Only, and Adjusting for Age, Sex, Time to Catheter Infection and Insertion Site **eTable 5.** Multivariable Logistical Models Accounting for Different Dwell Times (and Using Dwell Times ≤3 Days as Reference) **eTable 6.** Microbiological Etiology of Bloodstream Infections Associated With Peripheral Venous Catheters (PIVC-BSI) **eFigure.** Inverse Probability Treatment Weighted OR for PIVC-BSI for Different Dwell Times

Supplement 2.
Data Sharing Statement
