# Supplementary material for: Dwell Time and Risk of Bloodstream Infection With Peripheral Intravenous Catheters
Source: JAMA Netw Open. 2025 Apr 24;8(4):e257202. doi: 10.1001/jamanetworkopen.2025.7202 (PMC12022809; doi:10.1001/jamanetworkopen.2025.7202)
Supplement: Supplement 1. — eMethods. eTable 1. Multivariable Logistical Models Adjusting for Age, Sex, Time to Catheter Infection and Insertion Site eTable 2. Multivariable Logistical Models Accounting for Different Dwell Times eTable 3. Inverse Probability Treatment Weighted Logistical Regression Models for Different Dwell Times eTable 4. Multivariable Logistical Models Considering the First Catheter Only, and Adjusting for Age, Sex, Time to Catheter Infection and Insertion Site eTable 5. Multivariable Logistical Models Accounting for Different Dwell Times (and Using Dwell Times ≤3 Days as Reference) eTable 6. Microbiological Etiology of Bloodstream Infections Associated With Peripheral Venous Catheters (PIVC-BSI) eFigure. Inverse Probability Treatment Weighted OR for PIVC-BSI for Different Dwell Times [file jamanetwopen-e257202-s001.pdf]

Supplementary Online Content

Zanella MC, Catho G, Jackson H, et al. Dwell time and risk of bloodstream infection with peripheral intravenous catheters. *JAMA Netw Open.* 2025;8(4):e257202. doi:10.1001/jamanetworkopen.2025.7202

eMethods.

eTable 1. Multivariable Logistical Models Adjusting for Age, Sex, Time to Catheter Infection and Insertion Site

eTable 2. Multivariable Logistical Models Accounting for Different Dwell Times

eTable 3. Inverse Probability Treatment Weighted Logistical Regression Models for Different Dwell Times

eTable 4. Multivariable Logistical Models Considering the First Catheter Only, and Adjusting for Age, Sex, Time to Catheter Infection and Insertion Site

eTable 5. Multivariable Logistical Models Accounting for Different Dwell Times (and Using Dwell Times ≤3 Days as Reference)

eTable 6. Microbiological Etiology of Bloodstream Infections Associated With Peripheral Venous Catheters (PIVC-BSI)

eFigure. Inverse Probability Treatment Weighted OR for PIVC-BSI for Different Dwell Times

This supplementary material has been provided by the authors to give readers additional information about their work.

© 2025 Zanella MC et al. *JAMA Network Open.*

eMethods

Propensity score

As this was an observational study, the dwell time was not randomized and hence there were some baseline patient and PVC characteristics which were different for the dwell times investigated. Therefore, we developed propensity scores aimed to predict the conditional probability that a given PIVC would have a dwell time of more than 3 days (versus 3 days or less ≤3 days). We included the following covariates (i.e., related to dwell time and PIVC-BSI): age, sex, insertion site, time from hospital admission to catheter insertion. The distribution of propensity scores was checked graphically between the two groups. An inverse probability of treatment weighting based on the propensity score was computed to create a pseudo-population in which the distribution for the probability to have a PIVC dwell time of ≤3 days or >3 days was comparable. Then, the effect of dwell time >3 days *versus* ≤3 days was estimated using logistical regression models, weighted by IPTW (PROC GLIMMIX procedure of SAS). We have performed similar models for other dwell times (>4 days versus ≤4 days, >5 days versus ≤5 days, >6 days versus ≤6 days).

© 2025 Zanella MC et al. *JAMA Network Open.*

eTable 1. Multivariable Logistical Models Adjusting for Age, Sex, Time to Catheter Infection and Insertion Site

	OR	95% CI	p-value
**Dwell time >3 days**	13.55	5.44 - 34.0	<.0001
**Age**	1.00	0.99 - 1.011	0.93
**Female**	0.32	0.22 - 0.60	0.0002
**Time from hospital admission to catheter insertion**	0.995	0.98 - 1.02	0.60
**Insertion site (reference: wrist)**			
Forearm	0.66	0.33 - 1.22	0.13
Arm	1.11	0.3 - 3.5	0.87
Antecubital fossa	0.77	0.2 - 1.88	0.40
Hand	0.33	0.11 - 0.88	0.012

Legend. OR: Odds ratio. CI: confidence interval.

© 2025 Zanella MC et al. *JAMA Network Open.*

eTable 2. Multivariable Logistical Models Accounting for Different Dwell Times

	OR	95% CI
**>3 *versus* ≤3 days***	13.55	5.35-33.995
**>4 *versus* ≤4 days***	8.53	4.47-16.28
**>5 *versus* ≤5 days***	5.38	3.23-8.96
**>6 *versus* ≤6 days***	7.63	4.57-12.74

Legend. OR: Odds ratio. CI: confidence interval.* To increase the readability of eTable 2 adjustment factors were not showed for each model (i.e., age, sex, time from hospital-admission to catheter insertion, insertion site).

© 2025 Zanella MC et al. *JAMA Network Open.*

eTable 3. Inverse Probability Treatment Weighted Logistical Regression Models for Different Dwell Times

	**OR**	**95% CI**
**>3 *versus* ≤3 days**	11.06	4.63-26.42
**>4 *versus* ≤4 days**	7.97	4.09-15.52
**>5 *versus* ≤5 days**	5.47	3.31-9.05
**>6 *versus* ≤6 days**	7.77	4.78-12.64

Legend. OR: Odds ratio. CI: confidence interval. Covariates included in the propensity score were: age, sex, insertion site, time from hospital admission to catheter insertion.

© 2025 Zanella MC et al. *JAMA Network Open.*

eTable 4. Multivariable Logistical Models Considering the First Catheter Only, and Adjusting for Age, Sex, Time to Catheter Infection and Insertion Site

	**OR**	95% CI
**Dwell-time>3 days**	16.259	4.86-54.46
**Female**	0.29	0.12-0.67
**Age**	1.00	0.98-1.02
**Time from hospital admission to catheter insertion**	0.98	0.89-1.06
**Insertion site (reference: wrist)**		
Forearm	0.67	0.18-1.20
Arm	1.13	0.13-9.42
Antecubital fossa	0.67	0.19-2.40
Hand	0.28	0.08-1.01

© 2025 Zanella MC et al. *JAMA Network Open.*

eTable 5. Multivariable Logistical Models Accounting for Different Dwell Times (and Using Dwell Times ≤3 Days as Reference)

	OR	95% CI
**Reference ≤3 days**		
**4 days**	6.02	1.91-19.04
**5 days**	11.86	4.34-32.39
**≥6 days**	22.06	8.48-57.39
**Female**	0.33	0.18-0.60
**Age**	1.00	0.99-1.01
**Time from hospital admission to catheter insertion**	0.99	0.97-1.02
**Insertion site (reference: wrist)**		
Forearm	0.60	0.29-1.21
Arm	1.12	0.35-3.59
Antecubital fossa	0.63	0.23-1.73
Hand	0.29	0.11-0.80

Legend. OR: Odds ratio. CI: confidence interval.

© 2025 Zanella MC et al. *JAMA Network Open.*

eTable 6. Microbiological Etiology of Bloodstream Infections Associated With Peripheral Venous Catheters (PIVC-BSI)

	Dwell time ≤4 days	Dwell time >4 days	p-value
***Achromobacter* spp**	0 (0)	1 (2.0)	0.33
**CoNS or other skin commensals**	7 (58.3)	25 (51.0)	
***Enterobacter* spp**	0 (0)	4 (8.2)	
**Fungi**	0 (0)	2 (4.1)	
***Klebsiella* spp**	2 (16.7)	2 (4.1)	
**MRSA**	0 (0)	3 (6.1)	
**MSSA**	1 (8.3)	7 (14.3)	
Pseudomonas aeruginosa	0 (0)	3 (6.1)	
Serratia marcescens	1 (8.3)	0 (0)	
Sphingomonas paucimobilis	0 (0)	1 (2.0)	
**Polymicrobial**	1 (8.3)	1 (2.0)	

Legend. CoNS: Coagulase negative Staphylococci. MRSA: Methicillin-resistant *Staphylococcus aureus*. MSSA: Methicillin susceptible *Staphylococcus aureus*. Among 12 PIVC-BSIs of catheters with a dwell time ≤ 4 days 3 (25%) PVC-BSIs were due to Gram negative bacteria. Among 49 PIVC-BSIs of catheters with a dwell-time >4days 11 (22.4%) were due to Gram negative bacteria.

© 2025 Zanella MC et al. *JAMA Network Open.*

eFigure. Inverse Probability Treatment Weighted OR for PIVC-BSI for Different Dwell Times

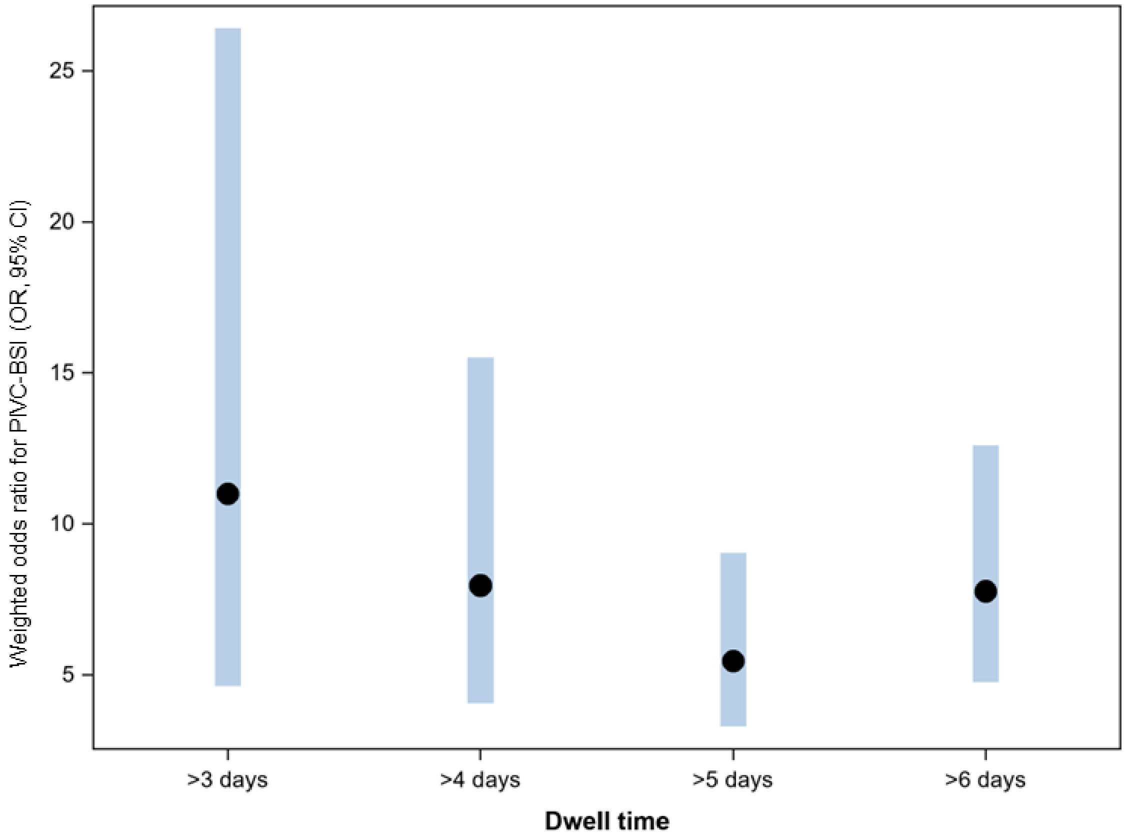

Legend. OR: Odds ratio. CI: confidence interval. PIVC: Peripheral venous catheter. BSI: Bloodstream infection. X-axis: References were ≤3 days, ≤4 days, ≤5 days and ≤6 days, respectively.

© 2025 Zanella MC et al. *JAMA Network Open.*
