# Supplementary material for: Dwell Time and Risk of Bloodstream Infection With Peripheral Intravenous Catheters
Source: JAMA Netw Open. 2025 Apr 24;8(4):e257202. doi: 10.1001/jamanetworkopen.2025.7202 (PMC12022809; doi:10.1001/jamanetworkopen.2025.7202)
Supplement: Supplement 2. — Data Sharing Statement [file jamanetwopen-e257202-s002.pdf]

Data Sharing Statement

Zanella. Dwell Time and Risk of Bloodstream Infection With Peripheral Intravenous Catheters. *JAMA Netw Open*. Published April 24, 2025. doi:10.1001/jamanetworkopen.2025.7202

Data
Data available: No
